# Phase Variation of NadA in Invasive Neisseria meningitidis Isolates Impacts on Coverage Estimates for 4C-MenB, a MenB Vaccine

**DOI:** 10.1128/JCM.00204-18

**Published:** 2018-08-27

**Authors:** Luke R. Green, Jay Lucidarme, Neelam Dave, Hannah Chan, Stephen Clark, Ray Borrow, Christopher D. Bayliss

**Affiliations:** a Department of Genetics and Genome Biology, University of Leicester, Leicester, United Kingdom; b Meningococcal Reference Unit, Public Health England, Manchester Royal Infirmary, Manchester, United Kingdom; c National Institute for Biological Standards and Control, Potters Bar, United Kingdom; Brigham and Women's Hospital

**Keywords:** 4C-MenB, Bexsero, MATS, NadA, Neisseria meningitidis, phase variation

## Abstract

A recombinant NadA protein is one of the four major protective antigens of 4C-MenB (Bexsero), a vaccine developed for serogroup B Neisseria meningitidis (MenB). The meningococcal antigen typing system (MATS) is utilized as a high-throughput assay for assessing the invasive MenB strain coverage of 4C-MenB. Where present, the *nadA* gene is subject to phase-variable changes in transcription due to a 5′TAAA repeat tract located in a regulatory region. The promoter-containing intergenic region (IGR) sequences and 5′TAAA repeat numbers were determined for 906 invasive meningococcal disease isolates possessing the *nadA* gene. Exclusion of the 5′TAAA repeats reduced the number of IGR alleles from 82 to 23. Repeat numbers were associated with low and high levels of NadA expression by Western blotting and enzyme-linked immunosorbent assay (ELISA). Low-expression repeat numbers were present in 83% of 179 MenB isolates with NadA-2/3 or NadA-1 peptide variants and 68% of 480 MenW ST-11 complex isolates with NadA-2/3 peptide variants. For isolates with vaccine-compatible NadA variants, 93% of MATS-negative isolates were associated with low-expression repeat numbers, whereas 63% of isolates with MATS relative potency (RP) scores above the 95% confidence interval for the positive bactericidal threshold had high-expression repeat numbers. Analysis of 5′TAAA repeat numbers has potential as a rapid, high-throughput method for assessing strain coverage for the NadA component of 4C-MenB. A key application will be assessing coverage in meningococcal disease cases where confirmation is by PCR only and MATS cannot be applied.

## Abstract (precis)

A recombinant NadA protein is one of the four major protective antigens of 4C-MenB (Bexsero), a vaccine developed for serogroup B Neisseria meningitidis (MenB). The meningococcal antigen typing system (MATS) is utilized as a high-throughput assay for assessing the invasive MenB strain coverage of 4C-MenB.

## INTRODUCTION

Reverse vaccinology identified Neisseria adhesin A (NadA) as a potent immunogen for eliciting antibody-mediated protection against *nadA^+^* serogroup B (MenB) strains of Neisseria meningitidis (1). Recombinant NadA was incorporated into 4C-MenB, a multicomponent MenB vaccine (2), and was shown to elicit protective serum bactericidal antibody (SBA) responses (3). Introduction of this vaccine into the UK infant immunization schedule in September 2015 raised new opportunities for understanding both functionality and coverage by the component antigens of this vaccine.

Four clusters of homology-based variants (NadA-1, NadA-2/3, NadA-4/5, and NadA-6) have been described for NadA peptide sequences (4). The NadA-1 and NadA-2/3 variants have similar lengths and homologies (∼60 to 80%), while the NadA-4/5 and NadA-6 variants are shorter and divergent (∼20 to 40% similarity). 4C-MenB contains peptide 8, a NadA-2/3 variant. Assessments of strain coverage with the meningococcal antigen typing scheme (MATS) indicate that the vaccine antigen elicits protective responses against strains expressing NadA-1 and NadA-2/3 peptides. While the *nadA* gene was found in only 22.3% (235/1,052 isolates) of European MenB isolates (5), NadA is present in several hypervirulent clonal complexes (6), including the recently emerged hypervirulent serogroup W (MenW) ST-11 sublineage (7–11). Thus, the NadA component of 4C-MenB is important for reducing disease due to hypervirulent MenB and MenW clones (12).

Expression of NadA undergoes phase variation (PV) due to 5′TAAA repeats present upstream of the core promoter and to regulation by a repressor protein (NadR/NMB1843) and by Fur (13–15). Three levels of expression—high, intermediate, and low—were linked to periodic changes in repeat number in stationary-phase cultures (13–15). Binding of integration host factor (IHF) within part of the repeat tract alters the DNA topology and facilitates contacts between upstream activator/repressor sequences, including the growth phase regulatory region (GPR), and RNA polymerase bound to the core promoter (13). The regulatory sequences are predicted to bind both the RNA polymerase alpha chain and NadR. Repression by NadR is alleviated by 4-hydroxyphenylacetate (4HPA), a component of saliva, and unknown molecules present in stationary-phase culture and infected blood cultures (2). The repeat number is presumed to influence the DNA orientation and contacts between the regulators and RNA polymerase. In addition, on/off PV of NadA-4/5 and some NadA-6 alleles is mediated by intragenic poly(C) and poly(G) homopolymers, respectively.

Meningococcal strain coverage by 4C-MenB is assessed using MATS. However, this assay requires a bacterial culture, and hence coverage cannot be assessed for the ∼50% of invasive meningococcal disease cases that are confirmed by nonculture PCR without recovery of a viable organism (16). We report on the prevalences of specific NadA 5′TAAA repeat numbers in a large collection of meningococcal disease isolates and on the utility of repeat number as a determinant of strain coverage.

## MATERIALS AND METHODS

### Genome sequence acquisition.

The Meningitis Research Foundation Meningococcus Genome Library (MRF-MGL) database (which included all clinical isolates from England, Wales, and Northern Ireland obtained between July 2010 and October 2016 and was accessed in May 2017) was interrogated by data set analysis for “NadA_peptide,” “NEIS1969,” “NEIS0374”, and “igr_up_NEIS1969” at https://pubmlst.org/bigsdb?db=pubmlst_neisseria_isolates. Isolates were sorted by NadA variant by use of a search by sequence attributes.

### Repeat tract analysis.

All contigs for each invasive isolate were extracted from the MRF-MGL database and annotated by use of PROKKA (version 1.11), with MC58 as a reference genome. Annotated contigs were interrogated using PhasomeIt (http://hdl.handle.net/2381/39872) to determine repeat numbers. Interrupted *nadA* sequences not detected by PhasomeIt were aligned using ClustalW within BioEdit (version 7.2.5), and repeat numbers were counted manually. A subset of 119 isolates (see Table S1 in the supplemental material), covering a range of serogroups and repeat numbers, was examined experimentally in order to confirm repeat number observations from the *in silico* analysis of whole-genome sequences. The same DNA samples as those used for whole-genome sequencing (WGS) were subjected to PCR amplification with the following primers to enumerate repeat tract length: NadA-for-FAM, 5′-6-carboxyfluorescein (FAM)-TCGACGTCCTCGATTACGAAGGC-3′; and NadA-rev 5′-TGGCTGTGGTCAGTACTTTGGATGG-3′. Fluorescently labeled amplicons were subjected to GeneScan fragment size analysis on an ABI sequencer (15). Downstream analysis of fragment size data was performed using Peakscanner (v1.0), BioEdit (v7.2.5), and Microsoft Excel.

### Construction of ΔNadA isolate.

NadA was amplified from the bacterial chromosome of R001, a MenW ST-11 carriage isolate, and replaced with a kanamycin resistance cassette. NadA gene flanking regions were amplified from R001 genomic DNA by use of primers NadA_UFR_F (5′-CCTACCATCAAGGCAGACCG-3′), NadA_UFR_R (5′-CGCTGGGTTTATCGGGATCCTGTCAGGATATGGGTTGCCG-3′), NadA_DFR_F (5′-AGATGTCTAAAAAGGGATCCCGATAAAGAAACCGCAGCC-3′), and NadA_DFR_R (5′-TCTGTTCATCGGTAAGCGCAC-3′), while the kanamycin resistance cassette was amplified from plasmid DNA by use of primers NadA_Kan_F (5′-ACAGGATCCCGATAAACCCAGCGAACCATTTG-3′) and NadA_Kan_R (5′-CGGGGATCCCTTTTTAGACATCTAAATCTAGGTAC-3′). NEBuilder HiFi DNA assembly master mix (New England Biosciences) was used to assemble the construct at a 1:1:1 ratio, using the manufacturer's guidance. Meningococcal isolate R001 was naturally transformed as described previously (17).

### Enzyme-linked immunosorbent assay (ELISA).

A subset of 45 *nadA*-positive MenB and MenW meningococcal isolates, identified from the MRF-MGL database, were obtained from the Public Health England (PHE) Meningococcal Reference Unit (MRU) and contained a range of repeat numbers in the NadA 5′TAAA repeat tract (Table S2). Relative NadA expression levels were quantified using meningococcal whole-cell lysates and a polyclonal rabbit antiserum raised against NadA peptide 8 (H. Chan and I. Feavers, personal communication). NadA-negative isolates were included as controls. Single colonies were restreaked and grown overnight on brain heart infusion (BHI) agar plates containing 10% Levinthal's supplement. Cells were disrupted by resuspension in lysis buffer (50 mM Tris, pH 8, 300 mM NaCl, 2 mM EDTA, 1% Triton X-100) and heat treatment at 56°C for 30 min. The protein concentration was determined by use of a NanoDrop ND-1000 spectrophotometer. Cell lysate (1 μg/ml) in coating buffer (35 mM NaHCO_3_, 15 mM Na_2_CO_3_, pH 9.6) was added to multiple wells of a flat-bottomed 96-well plate and incubated overnight at 4°C. Wells were treated with blocking buffer (phosphate-buffered saline [PBS], 0.05% Tween 20, 1% bovine serum albumin [BSA]) for 1 h and then probed with NadA-specific antiserum diluted in blocking buffer for 2 h. Wells were washed with PBST (PBS, 0.05% Tween 20) and probed with a goat anti-rabbit–alkaline phosphatase conjugate for 1 h. Wells were washed three times with PBST, followed by incubation with a PNPP substrate solution (Merck) and determination of the optical density at 405 nm (OD_405_) by use of a plate reader (Biotek Eon 6).

### Western blotting.

Cells were disrupted by resuspension in 1× sodium dodecyl sulfate loading buffer. The protein concentration was determined by use of a NanoDrop ND-1000 spectrophotometer. Cell lysates (50 μg) were subjected to electrophoresis in 10% polyacrylamide gels and transferred to polyvinylidene difluoride membranes. Membranes were blocked overnight in TBST-milk (20 mM Tris, pH 8, 140 mM NaCl, 0.1% Tween 20, 5% milk) and probed with NadA-specific antiserum diluted in TBST-milk for 2 h. Membranes were washed in TBST and probed with a 1:2,000 dilution of a goat anti-rabbit IgG–horseradish peroxidase conjugate. Bound antibodies were visualized by use of an enhanced chemiluminescence kit (Biological Industries) and photographic film. Bands were quantified via Fiji (v1.49) and normalized relative to that for an Hsp60 (MA3-022; Thermo Scientific) loading control.

### Colony immunoblots.

Single colonies were resuspended in 100 μl PBS and serially diluted to 10^−6^. Dilutions (100 μl) were plated onto BHI agar with 10% Levinthal's supplement and incubated overnight at 37°C with 5% CO_2_. Agar plates were replica plated onto nitrocellulose membranes and blocked in PBST-milk and sodium azide (PBS, 0.05% Tween 20, 5% milk, 0.1% sodium azide) for 1 h. Subsequently, membranes were probed in PBST-milk with a NadA antiserum diluted 1:1,500 for 2 h and washed before treatment with PBST-milk with anti-rabbit IgG–alkaline phosphatase diluted 1:2,000. Antibodies were visualized by use of a 5-bromo-4-chloro-3-indolylphosphate–nitroblue tetrazolium solution. Single colonies, picked due to differential staining, were boiled and processed to produce lysates containing genomic DNA. Lysates were analyzed by GeneScan to determine the repeat number.

### MATS assays.

The MATS assay was performed as previously described (18). The positive bactericidal threshold (PBT) for NadA is 0.009 (95% confidence interval [CI], 0.004 to 0.019) (19) and was defined as the minimum level of relative potency (RP) (versus the reference strain 5/99) required for an isolate to be considered susceptible to killing in the human serum bactericidal antibody assay using pooled sera from infants immunized with 4C-MenB.

### Statistical tests.

Repeat number distributions were analyzed by a chi-square test of observed versus expected distributions. MATS RP values were analyzed by analysis of variance (ANOVA) in GraphPad.

## RESULTS

### NadA distribution in invasive meningococci.

The NadA distribution was examined for 3,168 meningococcal invasive disease isolates obtained in the United Kingdom between July 2010 and October 2016. Most isolates were *nadA* negative (2,087/3,168 isolates [65.9%]), while 963 (30.4%) were *nadA* positive and 118 (3.7%) were unannotated. Only 19% (179/963 isolates) of *nadA*-positive isolates were MenB strains with vaccine-compatible NadA peptide variants (i.e., NadA-2/3 and NadA-1).

Most *nadA*-positive isolates possessed NadA-2/3 variants (618 isolates [64.2%]), with smaller numbers having NadA-1 (148 isolates [15.4%]) or NadA-4/5 (197 isolates [20.5%]) variants (Table 1). Thus, 79.5% (766/963 isolates) of *nadA*-positive isolates had vaccine-compatible NadA variants. The NadA-2/3 variants were mainly observed within clonal complex 11 (CC11) isolates (85.8% [530/618 isolates]), of which 91% were in recently isolated serogroup W (480/530 isolates). NadA-2/3 variants were also highly prevalent in CC174 (100% [21/21 isolates]) and CC1157 (91% [19/21 isolates]) isolates, which were mainly of serogroups Y (19) and B (18), respectively. Most NadA-1 variants were found among CC32 isolates (88.5% [131/148 isolates]), of which 127 were serogroup B isolates, while most NadA-4/5 variants were observed in serogroup B, CC213 (B:CC213) isolates (85.8% [169/197 isolates]). An *in silico* analysis of NadA-4/5 variants indicated that a significant proportion did not express functional NadA (90.4% [178/197 isolates]) due to out-of-frame repeat numbers in a phase-variable poly(C) tract (e.g., 10 repeats in 107 isolates).

**TABLE 1:** NadA peptides in UK invasive disease isolates collected between July 2010 and October 2016^*a*^

NadA variant	Genogroup	Clonal complex	NadA peptide(s)	NadR allele(s)	NadA IGR(s)	No. of 5′TAAA repeats^*b*^	
						Phases 1 and 2	Phase 3
NadA-2/3 (618)	A	CC5	8	1	1		9
	B (35)	CC11 (7)	3 (5), 4, 6	1 (7)	1, 4 (6)	10, 14	9 (2), 12 (3)
		CC1157 (18)	0^*c*^ (18)	1 (16), 5 (2)	6 (16), ND (2)	5 (2)	6 (16)
		CC18	8	9	14		12
		CC213	0	1	6		6
		CC269	8	1	7		9
		CC461	3	6	4		12
		CC60	8	1	7		9
		ND (5)	0 (2), 8 (2), 146	1 (5)	1 (4), 6	7, 11	6 (2), 12
	C (46)	CC11 (42)	2, 3, 4, 6, 121 (29), 127 (9)	1 (41), 6	1, 4 (32), 13, ND (8)	10 (2), 11, 13 (2), 17, ND (4)	6, 9 (16), 12 (15)
		CC174 (2)	8 (2)	1 (2)	7 (2)		9
		CC8	7	1	1		12
		ND	121	1	4		12
	E	CC60	3	1	4		12
	NG (6)	CC11 (2)	3, 6	1, ND	1, 4		12, 18
		CC1157	0	1 (3)	6 (3)	5	6 (2)
		ND	8	ND	1		6
	W (509)	CC11 (480)	3, 6 (477), 130, 157	1 (447), 3 (2) 5 (5), 6 (2), 12 (4), 40 (7), 46, 101, 105, 159 (2), 210, 259, 272, ND (5)	1 (345), 9 (11), 17, 18, 19, 20, ND (120)	7 (6), 8 (23), 10 (23), 11 (21), 13 (18), 14 (8), 16 (4), 17 (13), 19, ND (47)	3, 6 (6), 9 (93), 12 (171), 15 (43), 18 (2)
		ND (29)	6 (29)	1 (29)	1 (26), 9 (2), 21	10 (2), 11, 13 (2), 14, 16, 19	6 (2), 9 (7), 12 (7), 15 (3)
	W/Y	CC174	8	5	7	11	
	Y (19)	CC11	6	1	1		12
		CC174 (18)	8 (17), 131	1 (14), 9, 12, 40, 46	7 (14), 15, ND (3)	11	6, 9 (3), 12 (13)
NadA-1 (148)	B (144)	CC32 (127)	0^*c*^ (5), 1 (91), 100 (26), 118, 137, 141, 158, 159	1 (11), 4 (66), 5 (10), 6 (3), 12 (3), 77 (19), 91, 96, 118 (2), 127, 136, 204, 206, 207, ND (3)	1, 3 (89), 5 (25), 12, 16, ND (10)	5 (33), 7, 8 (2), 11, 13, ND (2)	3, 6 (61), 9 (10), 12 (15)
		ND (17)	1 (17)	4 (14), 5, 6, 17	3 (15), ND (2)	5	6 (13), 9, 12, 15
	C (4)	CC32 (4)	1 (4)	4 (3), 5	5 (4)	4, 5 (3)	
NadA-4/5 (197)	B (194)	CC1157 (2)	0 (2)	40 (2)	11 (2)	5 (2)	
		CC213 (169)	0 (164), 21 (2), 79 (2), 122	1 (21), 2 (24), 3 (5), 5 (14), 6 (20), 9 (8), 12 (10), 15, 16, 46 (59), ND (6)	2 (152), 8, 10 (10), 22, 23, ND (4)	4, 5 (2), 7 (40), 8 (19), 10 (11), 11 (10), 13, 14, 16 (3), 17 (3), 19 (2), 20, 22, 23, ND (2)	6 (16), 9 (31), 12 (17), 15 (3), 18 (2), 21 (2)
		CC269 (3)	0 (3)	5, 9 (2)	2 (3)	7, 8	9
		CC32 (1)	21	77	8		12
		CC60 (1)	0 (1)	1	2		9
		ND (18)	0 (8), 21 (10)	1 (4), 3, 5 (2), 6, 9 (2), 12 (3), 46 (5)	2 (7), 8 (8), 10 ND (2)	7 (2), 8 (2), 10, 11 (2), 17 (2), ND (1)	6, 9 (3), 12 (4)
	W (2)	ND (2)	21 (2)	6 (2)	8 (2)	16	12
	Y	CC865	21	1	8		12

a Numbers of isolates are given in parentheses unless there was only one isolate. ND, no data.

b Phases 1 and 2, repeat numbers that increase in increments of three from 4 or 5 repeats; phase 3, repeat numbers that increase in increments of three from 3 repeats.

c Peptide allele 0, a frameshifted NadA allele or a pseudogene which is frameshifted, contains one or more stop codons, or is an insertion element.

### Variation within critical NadA intergenic regions.

The *nadA* intergenic region (IGR) is located between NMB1993 and *nadA*. This region, termed igr_up_NEIS1969, was extracted from 812 of 963 *nadA*-positive isolates and aligned to reveal homologous sequences (note that the other 151 isolates had incompletely assembled IGRs). A total of 82 *nadA* IGR alleles were identified, with lengths between 279 and 368 bp. These IGRs contain the 5′TAAA repeats, which are likely to vary in a manner independent of other types of allelic variation. On exclusion of the repeats, the number of unique IGR alleles was reduced to 23, and these alleles, termed the NadA IGRs, were numbered based on their prevalence within the MRF-MGL database.

**FIG 1:**
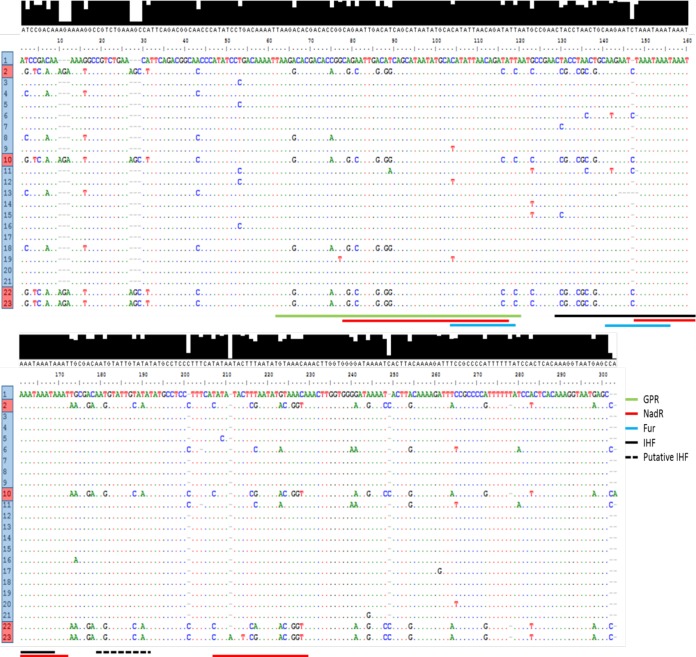
Variation in *nadA* IGR sequences. A set of 23 unique IGR sequences were observed after igr_up_NEIS1969 sequences extracted from Neisseria PubMLST were filtered after normalization of repeat numbers. Regulatory regions are shown as follows: green, growth phase regulatory region (GPR); red, NadR; blue, Fur; black, integration host factor (IHF). Histograms above the alignment give a visual representation of the homology of each base between IGR sequences. Sequences are placed in order of frequency within the MRF-MGL database, with 1 being the most prevalent and 23 the least. Clusters are signified by the color of the allele number, as follows: blue, C1; and red, C2.

Putative functional regions of the NadA IGR are shown in Fig. 1 (13). Two distinct clusters of IGRs (C1 and C2) were observed, with sequence variation in all critical regions. C1 was separated from C2 by a minimum of 56 differences that included both single nucleotide polymorphisms (SNPs) and indels. The C1 and C2 groups contained 19 and 4 IGRs, respectively, with the C2 IGRs (IGRs 2, 10, 22, and 23) being associated exclusively with NadA-4/5 alleles. C1 was present in 77% of isolates (621/812) with a NadA IGR. Variation was highest within the 58-bp GPR and the 22-bp NadR binding region 2.

Linkage of IGRs and specific NadA variants was detected, with predominant associations between IGR 3 and NadA-1 (77% [104/136 isolates]), IGR 1 and NadA-2/3 (79% [382/485 isolates]), and IGR 2 and NadA-4/5 (85% [163/191 isolates]) (Table 1). However, the number of unique IGR alleles was higher for NadA-2/3 isolates (57% [13/23 alleles]) than for the NadA-1 (22% [5/23 alleles]) or NadA-4/5 (26% [6/23 alleles]) isolates. Furthermore, some clonal complexes were correlated with specific combinations of NadA peptide and IGR allele. Thus, W:CC11 strains almost exclusively possessed NadA-2/3 variants and IGR allele 1 (72% [345/480 isolates]), while B:CC1157 isolates mostly encoded NadA-2/3 variants with IGR allele 6 (80% [16/20 isolates]). Additionally, 70% (89/127 isolates) of B:CC32 isolates harbored NadA-1 variants with IGR allele 3, and 89% (152/170 isolates) of B:CC213 isolates possessed NadA-4/5 with IGR allele 2 (Table 1).

As variation in the NadR binding region may influence NadR binding, there is potential for covariance of the *nadA* IGR and the NadR peptide. However, no associations were detected, with NadR allele 1 being linked to 21 NadA IGRs and NadR allele 2 linked to 7 NadA IGRs (Table 1).

### Specific 5′TAAA repeat lengths are prominent in UK clinical isolates.

NadA 5′TAAA repeat numbers were determined by bioinformatic analyses of genome sequence data. The repeat tract was fully assembled and characterized for 94.2% of 962 NadA-positive isolates (Table 1) (tracts were incomplete for the other 55 isolates). Identical repeat numbers were observed for genomic and GeneScan analyses of 100 of a subset of 109 isolates, giving an accuracy of 91.7% for the genomic analysis (Table S1). For six of the discordant isolates, genomic data predicted 17 or 19 repeats, while tracts of 14, 16, or 20 repeats were found by GeneScan. These differences may reflect variation occurring during regrowth of bacterial strains due to the high mutability in long tetranucleotide repeat tracts (20, 21) or an inability of Illumina sequencing technologies to accurately sequence repetitive tracts of >67 bp.

**FIG 2:**
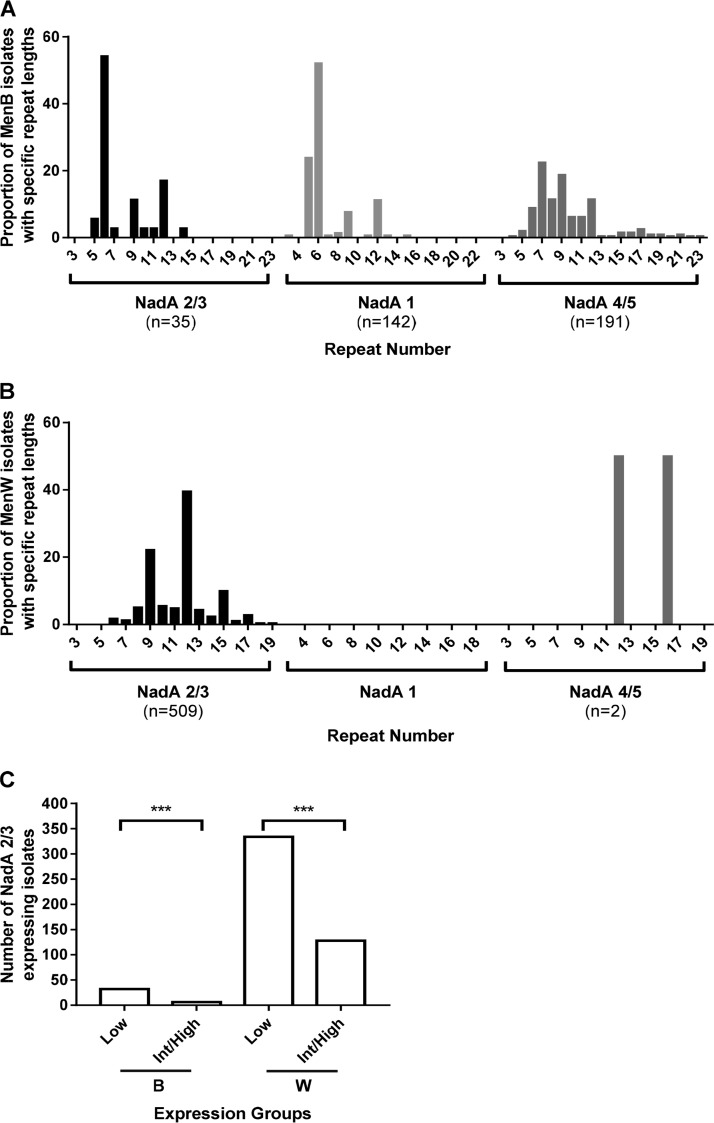
NadA 5′TAAA repeat tract lengths of 6, 9, and 12 are predominant in UK clinical meningococcal isolates. Whole-genome sequences of invasive disease meningococcal isolates isolated in the United Kingdom between July 2010 and October 2016 were extracted from the MRF-MGL database. Sequences were analyzed using PhasomeIt to extract and analyze *nadA* 5′TAAA repeat tracts. Repeat numbers were classified into the following expression classes: low, 3, 5, 6, 9, 12, 15, 18, and 21 repeats; and intermediate/high, 7, 8, 10, 11, 13, 14, 16, 17, 19, and 20 repeats. (A) MenB isolates separated by NadA variant. (B) MenW isolates separated by NadA variant. (C) MenB and MenW isolates expressing NadA-2/3 peptides grouped by predicted expression based on repeat number. Data were analyzed by the chi-square test (***, *P* ≤ 0.001).

Tracts of 6, 9, and 12 repeats were consistently observed with NadA-2/3 alleles (60.7% [375/618 isolates]), while 6 repeats were predominant in MenB isolates with NadA-1 alleles (51.4% [78/144 isolates]) (Fig. 2). Contrastingly, 7 was the most frequent repeat number among MenB isolates with NadA-4/5 alleles, and these isolates also exhibited a larger range of repeat numbers than that for MenB:NadA-2/3 alleles (4 to 23 and 5 to 14, respectively). Higher repeat numbers were consistently observed in MenW:NadA-2/3 isolates than in MenB:NadA-2/3 isolates, with the modal repeat number being 12 (35.0% [178/509 isolates]) and 6 (54.3% [19/35 isolates]), respectively (Fig. 2).

### Alternating repeat lengths lead to periodic expression in different NadA variants.

**FIG 3:**
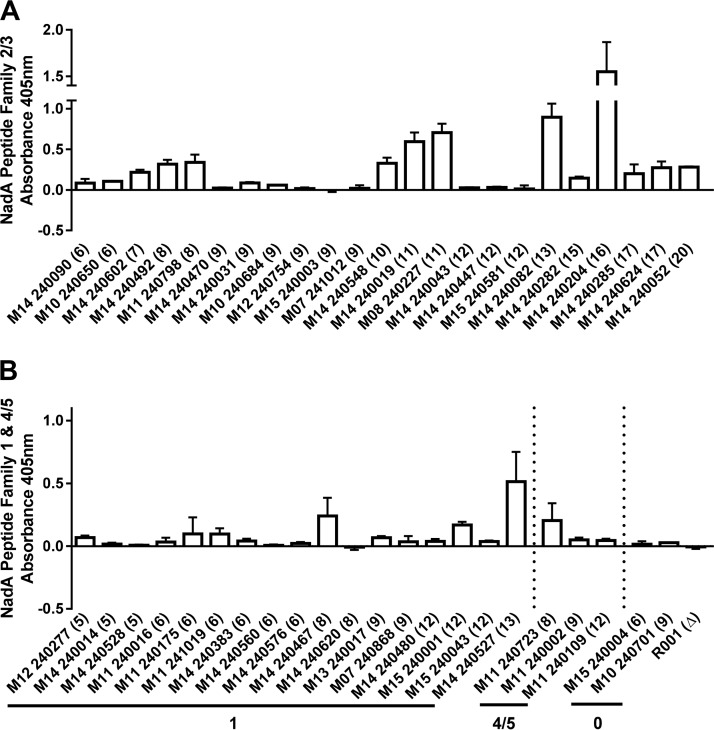
Periodic expression of NadA-2/3, NadA-1, and NadA-4/5 peptide variants. Subsets of meningococcal isolates were selected based on repeat number. Repeat numbers were confirmed by GeneScan analysis and sequencing. NadA expression was determined by ELISA. (A) NadA expression in isolates expressing NadA-2/3 peptides. (B) NadA expression in isolates expressing either NadA-1 or NadA-4/5 peptides. A value of 0 represents isolates not expressing a NadA peptide due to stop codons in the reading frame or a full deletion of the gene. Numbers in parentheses represent the 5′TAAA repeat number. Each bar represents three biological replicates; data are means and standard deviations (SD).

NadA expression was measured by ELISA and Western blotting for MenB:CC32, MenB:CC213, MenB:CC269, MenB:CC1157, and MenB:CC11/MenW:CC11 isolates with differing repeat numbers and NadA variants. Periodic expression was observed in both MenB and MenW strains; however, the pattern varied depending on the NadA variant. MenB:CC11 and MenW:CC11 isolates harboring NadA-2/3 had similar patterns of periodic expression, with 6, 9, 12, and 15 repeats producing low levels of expression (Fig. 3A; Fig. S1A and S2A). Both 17 and 20 repeats also resulted in low NadA expression, suggesting that the periodicity may be altered by these long repeat tracts. All other repeat numbers produced higher levels of expression, with 7 and 10 repeats producing intermediate levels of expression compared to the high expression levels with 8 and 11 repeats, respectively. Application of this NadA expression pattern to all invasive isolates with NadA-2/3 peptides indicated that low-expression repeat numbers occurred in a significantly larger number of isolates than the high-expression repeat numbers for both the MenB and MenW serogroups (*P* < 0.0001 and *P* < 0.0001, respectively; chi-square test) (Fig. 2C).

Analysis of MenB:CC32 isolates with NadA-1 alleles found that 5, 6, 9, and 12 5′TAAA repeats were associated with low levels of expression compared to the high expression levels in comparator variants with 8 and 13 repeats (Fig. 3B; Fig. S1B and S2B). Two isolates, M11 240016 and M14 240620, with 6 and 8 repeats, respectively, demonstrated no expression, similar to isolates with a frameshifted *nadA-1* allele and with a full *nadA* deletion (M10 240701 and R001*ΔnadA*, respectively) (Fig. 3B). Both of these MenB isolates possess peptide 100, which is an atypical NadA-1 allele with an 86-amino-acid C-terminal extension (Fig. S3). For NadA-4/5, isolates possessing a *nadA* allele with an intragenic poly(C) tract of 5 repeats (too short to mediate efficient PV [22]) exhibited low-level expression with 9 and 12 5′TAAA repeats and high expression with 8 5′TAAA repeats.

For isolates with intermediate- and high-expression repeat tract numbers, ELISA suggests that there is a correlation between increases in NadA expression and repeat number (Fig. 3). For example, M11 240798 exhibited high expression with 8 repeats (0.339), while M08 240227, also considered a high expressor and possessing 11 repeats, had a 2-fold higher level of expression (0.706). Similarly, NadA-2/3 isolates with 7, 10, 13, and 16 repeats exhibited increasing levels of NadA expression.

Two phase variants, with 11 and 13 repeats, were selected from an isogenic MenW:CC11 isolate, M14 240043 (with 12 repeats); these variants exhibited higher NadA expression than that of the parent strain (Fig. S4). Moreover, these phase variants produced expression levels similar to those in other MenW:CC11 isolates with identical repeat numbers (data not shown).

### Associations between 5′TAAA repeat numbers and MATS relative potency values.

**FIG 4:**
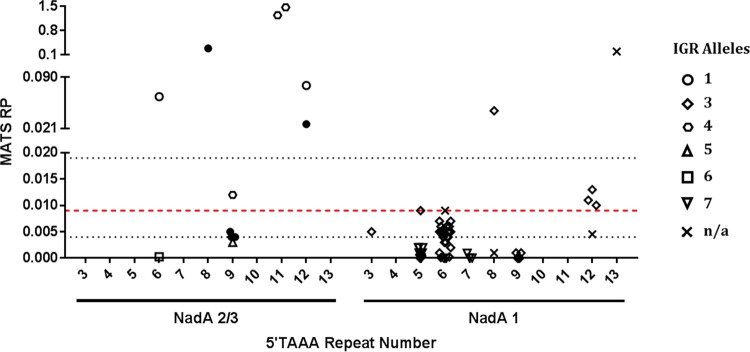
NadA repeat number is a potential correlate of protection by 4C-MenB. Subsets of MenB and MenW isolates were analyzed by the NadA MATS assay. Relative potency scores were compared against NadA repeat numbers and IGR types. In each case, isolates were separated by NadA variant. Scores above or equal to the PBT (red line) of 0.009 were considered protective. Black lines represent 95% confidence intervals for the PBT. Each symbol represents a different IGR type. Open symbols, MenB isolates; filled symbols, MenW isolates. n/a, not available.

NadA MATS RP values were generated for 68 MenB and 6 MenW isolates during enhanced surveillance of 4C-MenB by Public Health England. To determine how the repeat number impacts on MATS values, the NadA RP value was compared to the repeat number for each isolate (Fig. 4; Table S3). All NadA-4/5-possessing CC213 isolates (*n* = 3) had RP values below the PBT and were excluded from further analysis.

Only 14 of 71 NadA-1- or NadA-2/3-harboring isolates had RP values above the PBT, the threshold for coverage by 4C-MenB (Fig. 4). Among these 14 MATS-positive isolates were 2 MenB:CC11:NadA-2/3 isolates with 11 repeats, 1 MenW:CC11:NadA-2/3 isolate with 8 repeats, and 2 MenB:CC32:NadA-1 isolates with 8 or 13 repeats. All these repeat numbers were associated with high NadA expression. The remaining 9 MATS-positive isolates included 5 isolates with 12 repeats (1 MenW:CC11:NadA-2/3, 1 MenB:unassigned:NadA-2/3, and 3 MenB:CC32:NadA-1 isolates), 1 with 9 repeats (MenB:CC11:NadA-2/3), 1 with 5 repeats (MenB:CC32:NadA-1), and 2 with 6 repeats (1 MenB:unassigned:NadA-2/3 and 1 MenB:unassigned:NadA-1 isolate). Three of these isolates exhibited high RP values (0.027, 0.064, and 0.079), while the other 6 had RP values above the PBT but within the 95% confidence intervals (Fig. 4; Table S3). Among the 57 MATS-negative isolates, 53 had repeat numbers (i.e., 3, 5, 6, 9, or 12 repeats) associated with low NadA expression (Fig. 4). The 4 exceptions had 7 or 8 repeats, associated with intermediate or high expression, respectively. However, the isolate with 8 repeats had the atypical NadA peptide 100, which may not be presented properly on the cell surface. Thus, MATS positivity is associated with high/intermediate-expression repeat numbers, with a sensitivity of 5/14 isolates (36%), and this increases to 5/8 isolates (63%) if isolates with NadA RP values within the PBT 95% confidence intervals are removed. Conversely, MATS-negative isolates can be associated with low-expression repeats, with a high specificity of 53/57 isolates (93%).

If associations are considered by repeat number, all isolates with 3 or 7 repeats (*n* = 4) and the majority with 5 (94% [15/16 isolates]), 6 (93% [25/27 isolates]), or 9 repeats (91% [10/11 isolates]) had RP values below the PBT. Conversely, 8, 11, and 13 repeats were usually associated with RP values above the PBT (83% [5/6 isolates]). Surprisingly, most isolates with 12 repeats (83% [5/6 isolates]) produced RP values above the PBT. For highly vaccine-homologous NadA-2/3 peptides, intermediate/high-expression repeat numbers (8 and 11) had MATS RP values that were significantly higher than those for both the 6/9- and 12-repeat number groupings (Fig. 4; Fig. S5A).

Alternative comparisons were made for MATS RP values versus NadA IGR alleles or peptides. Most of the seven tested IGRs were associated with RP values above and below the PBT (Fig. 4). Significant differences in MATS RP values were not detected for either NadA-2/3 IGR alleles or NadA peptides, apart from IGR 4 (*n* = 3) and peptide 3 (*n* = 2) (Fig. S5B and C); however, the last two comparisons are confounded by two isolates having the peptide 3/IGR 4 combination and 11 repeats, a high-expression repeat number (Table S3). Overall, we did not detect associations of NadA IGR or peptide sequence *per se* (i.e., without repeat variation) with the average RP value, indicating that repeat number is the major determinant of MATS outputs.

To confirm the associations between NadA repeat numbers and MATS values, we analyzed a set of 19 MATS-tested isolates by ELISA and Western blotting (Fig. 3; Fig. S2). All four isolates with intermediate/high-expression repeat numbers and MATS RP values of >0.045 exhibited higher expression than that in isolates with lower repeat numbers. Isolate M14 240467, which encodes peptide 100 and has 8 5′TAAA repeats, exhibited low expression, suggesting that the peptide reduces expression or immune detection.

To address whether observed differences were due to mixed populations containing highly and lowly NadA-expressing variants, we artificially mixed isolates with 13 repeats or 11 repeats with an isolate containing 12 repeats (Fig. S6). We observed that the presence of 20% or more of the high expressor elevated the observed ELISA values 3- to 5-fold over those for the low expressor alone. Conversely, a lysate containing 10% of the high expressor showed an expression level similar to that in a lysate derived from only the low expressor (Fig. S6).

## DISCUSSION

Introduction of 4C-MenB into the UK national infant immunization schedule led to commencement of enhanced surveillance to assess MenB strain coverage, with MATS as a critical component assay. However, ∼50% of invasive meningococcal disease cases are confirmed by PCR only and hence cannot be assessed by MATS, as this assay requires a viable culture (16). Next-generation sequencing of all UK meningococcal disease isolates has enabled new bioinformatic approaches to strain analysis. Here we investigate the utility of the NadA 5′TAAA repeat number for predicting 4C-MenB-related NadA peptide strain coverage.

Previously, periodic repeat-mediated differences in NadA expression were detected by Western blotting and transcriptional analyses (13, 14). Using ELISAs and Western blotting, we confirmed that NadA-2/3 peptides of both MenB and MenW isolates exhibited low expression levels when 6, 9, or 12 repeats were present (13, 14). Similarly, NadA-1-positive isolates with 5, 6, 9, or 12 repeats were associated with low expression. Five repeats had not previously been tested, and our results suggest that short repeat tracts hinder efficient NadA expression. These associations were utilized for analysis of genomic data from a large set of UK meningococcal clinical isolates. Overall, we observed that 70% of Nad-2/3 (433/618 variants) and NadA-1 (103/148 variants) variants were associated with low-expression repeats, and this increased to 92% (136/148 variants) for the NadA-1 alleles upon inclusion of 5 repeats (Table 1; Fig. 1). For MenB strains, the target for 4C-MenB, 83% of MenB:NadA-2/3 (29/35 isolates) and 85% of MenB:NadA-1 (122/144 isolates) disease isolates had low-expression-associated repeat numbers. Importantly, 68% (326/480 isolates) of NadA-2/3-containing MenW ST-11 complex strains also had low-expression repeat numbers.

An analysis of MATS outputs found that the NadA 5′TAAA repeat number, but not the NadA IGR (excluding the repeat tract) or peptide, was strongly correlated with coverage prediction. Thus, low/intermediate-expression repeat numbers (i.e., 5, 6, or 9) were associated with RP values below the PBT, while high-expression repeats (i.e., 8, 11, or 13) correlated with values above the PBT. Tracts of 12 repeats were problematic, as high RP values were detected for this low-expression repeat number. Additionally, an isolate with 6 repeats (M10 240650; NadA peptide 8; NadA IGR 1) had a high RP. Discrepancies may arise due to heterogeneous stock cultures resulting in unrepresentative mixtures of phase variants being used for MATS versus genomic DNA preparation. Alternatively, PV during culture preparation for MATS may result in mixed populations containing small numbers of highly NadA-expressing cells that artificially elevate the RP value, as evidenced in our ELISAs, whereby 20% or more of a high expressor elevated the observed NadA expression level of a low expressor (see Fig. S6 in the supplemental material). This phenomenon would occur more frequently with 12 as opposed to 9 repeats due to more switching of the longer tract. Interestingly, RP values for isolates with matching NadA peptides and IGRs but containing 12 repeats were 10 and 4 to 5 times lower than those for high expressors for variant 2/3 and variant 1 NadA alleles, respectively (Table S3). Note that RP values for 12-repeat variant 1 versus variant 2/3 alleles are probably lower due to low homology of these variants with the vaccine antigen. Further testing is required to determine whether isolates with 12 repeats should be designated covered or “at risk” of not being covered (i.e., equivalent to above or below the PBT, respectively).

Our analysis indicates that two strains with either high (e.g., 8 or 11)- or low (e.g., 9)-expression-associated repeat numbers but the same NadA peptide/IGR allele combination will in the former case exhibit a protection-associated RP value and in the latter a nonprotective value. Similar alterations in RP value may also occur during a meningococcal infection due to PV between high and low NadA expression. Assessing the coverage of strains and disease isolates is therefore arbitrarily influenced by a repeat number that can change during isolation or manipulation of meningococci. Performing MATS or SBA assays on defined meningococcal phase variants with high and low expression states (e.g., 8 and 9 5′TAAA repeats) will establish how the expression level influences outputs from these assays. Similarly, comparisons of differing NadA peptides should utilize high-expression repeat numbers (e.g., 8 5′TAAA repeats) in order to properly determine how vaccine antigen homology influences the correlates of protection. Determination of the NadA repeat number by PCR amplification of meningococcal DNA extracted from clinical specimens, as successfully applied to the phase-variable hemoglobin receptors (23), may provide a method for assessing strain coverage in disease cases lacking a viable culture and control for changes in repeat number during strain isolation in situations where cases are confirmed by both PCR and culture.

Our data rely upon *in vitro* predictions of the NadA expression state. However, NadA expression may be upregulated *in vivo* due to antagonism of NadR repression (2). There is also differential expression of NadA during *in vitro* growth in rich media, with higher expression in stationary phase than in the logarithmic phase of growth (13). Thus, predictions of coverage via IGR, repeat tract, and peptide sequence, as with MATS RP values, are conservative, as isolates with NadA PV states with low *in vitro* expression may still have sufficient NadA expression for killing by 4C-MenB-elicited antibodies during actual infections (24).

Herein we describe a novel method for predicting coverage of the NadA component of 4C-MenB based on the *nadA* repeat number. We found that *nadA* expression in MenB and MenW clinical isolates obtained since 2010 was significantly associated with low-PV expression states and that these PV states correlate with low RP values in the MATS assay, suggesting that repeat number is a useful predictor of coverage for vaccine-homologous NadA alleles. Together these data indicate that characterization of NadA peptides and repeat numbers can be used as a surrogate for prediction of vaccine coverage in nonculture cases of meningococcal disease.

## Supplementary Material

Supplemental file 1

Supplemental file 2
