# Supplementary material for: Phase Variation of NadA in Invasive Neisseria meningitidis Isolates Impacts on Coverage Estimates for 4C-MenB, a MenB Vaccine
Source: J Clin Microbiol. 2018 Aug 27;56(9):e00204-18. doi: 10.1128/JCM.00204-18 (PMC6113495; doi:10.1128/JCM.00204-18)
Supplement: Supplemental file 1 [file zjm999096083s1.pdf]

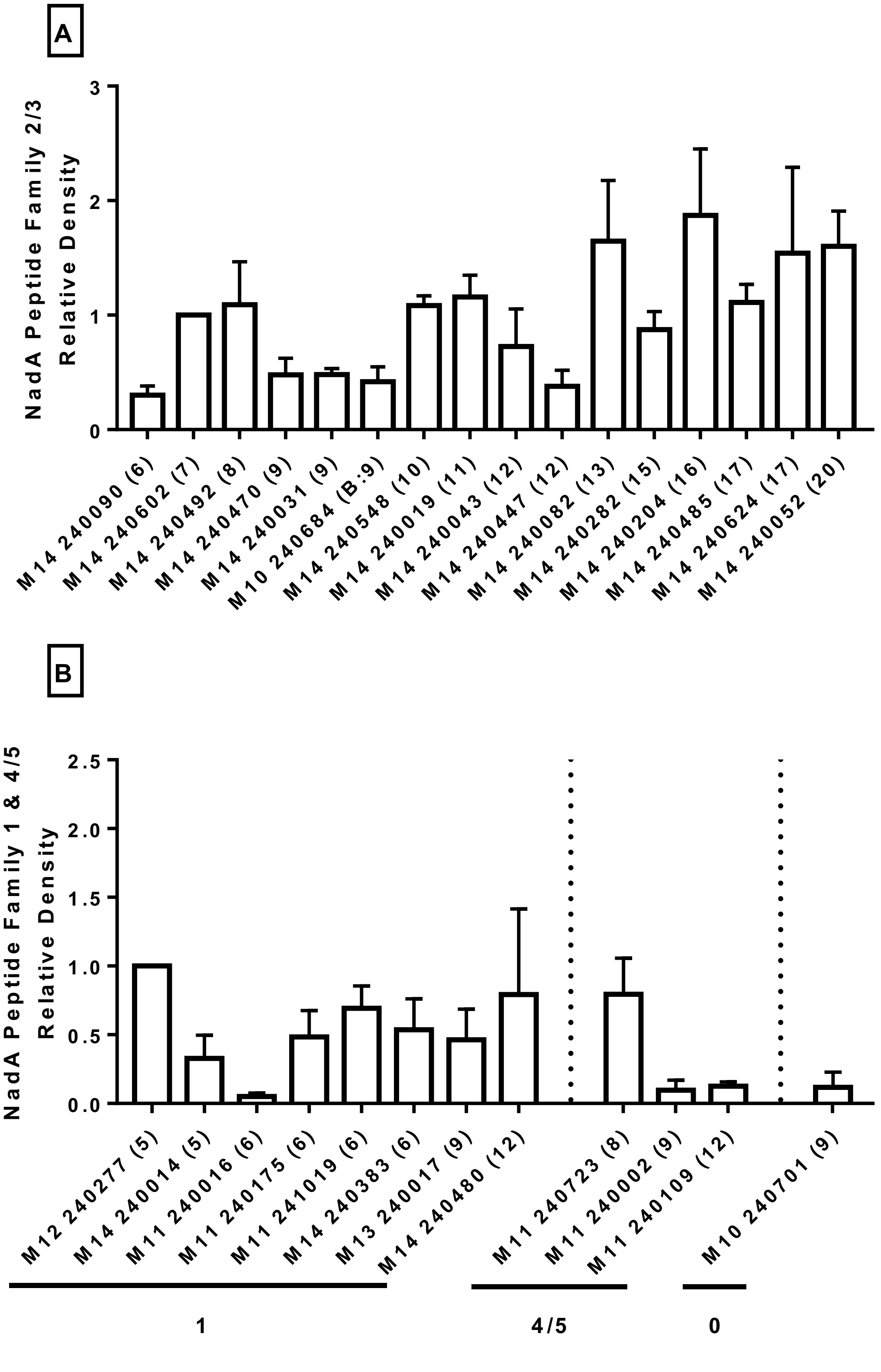

FIG. S1. Examination of the periodic expression of NadA-2/3, NadA-1 and NadA-4/5 peptide variants by Western blot analysis. Subsets of meningococcal isolates were selected based on repeat number. Repeat numbers were confirmed by GeneScan analysis and sequencing. NadA expression was determined by Western blot and quantified by densitometry. NadA bands were normalised against Hsp90 bands and then relative density was assessed against either (A) M14 240602 (MenW) or (B) M12 240277 (MenB).

(A) NadA expression in isolates expressing NadA-2/3 peptides. (B) NadA expression in isolates expressing either NadA-1 or NadA-4/5 peptides. 0 represents isolates not expressing a NadA peptide due to stop codons in the reading frame. Numbers in brackets represent the 5'TAAA repeat number. Each bar represents four biological replicates, mean $\pm$ SEM.

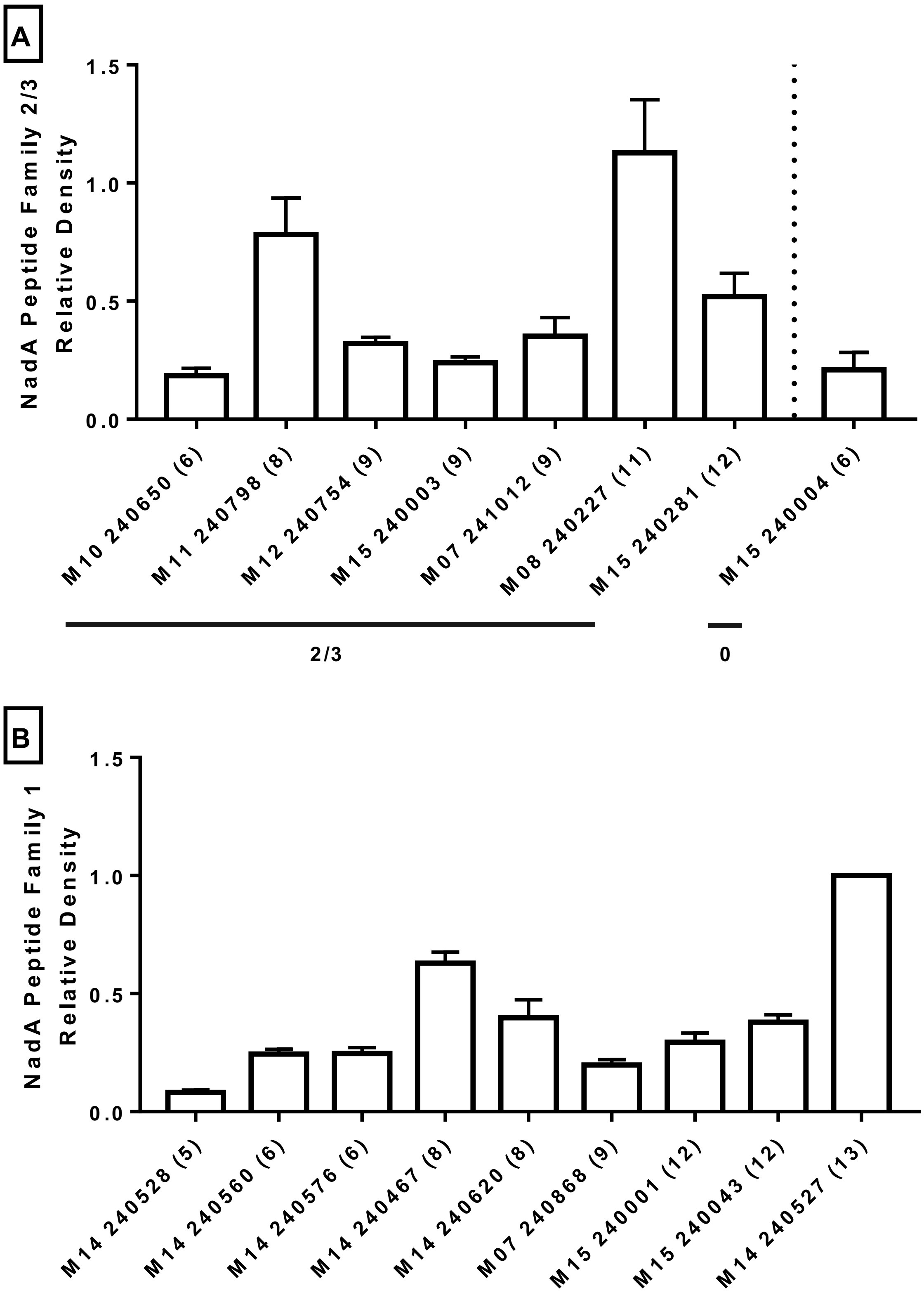

FIG. S2. Examination of NadA expression for MATS-tested NadA isolates by Western blot analysis. Subsets of meningococcal isolates were selected based on previous MATS testing and repeat number. Repeat numbers were confirmed by GeneScan analysis and sequencing. NadA expression was determined by Western blot and quantified by densitometry. NadA bands were normalised against Hsp90 bands and then relative density was assessed against M14 240527. (A) NadA expression in isolates expressing

NadA-2/3 peptides. (B) NadA expression in isolates expressing NadA-1 peptides. 0 represents isolates not expressing a NadA peptide due to stop codons in the reading frame. Numbers in brackets represent the 5'TAAA repeat number. Each bar represents three biological replicates, mean ± SEM.

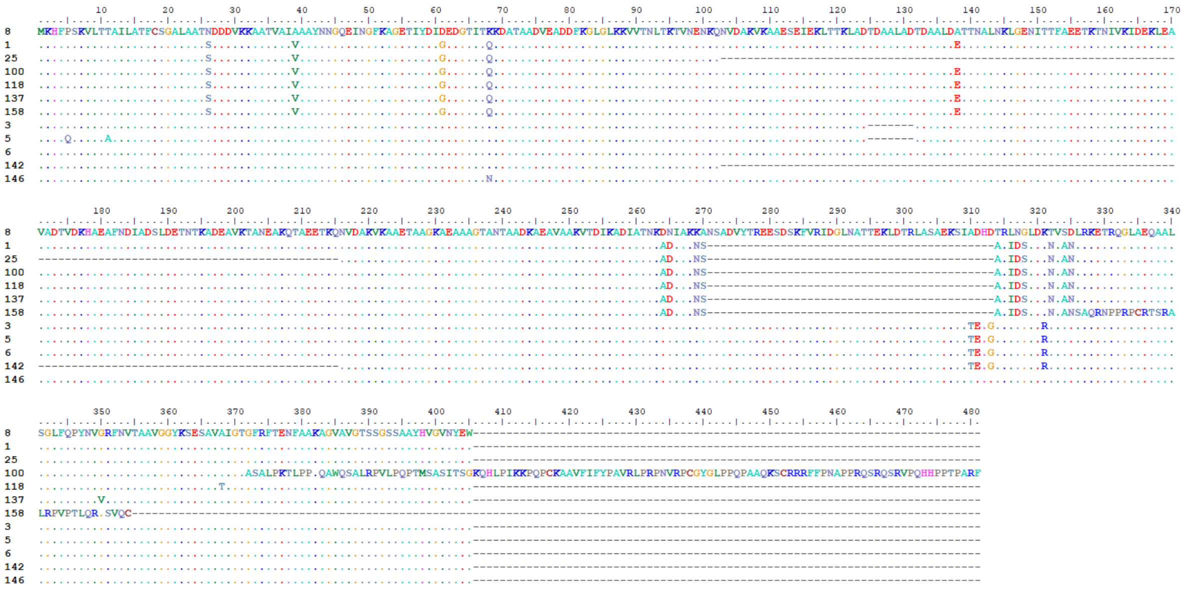

Fig. S3. Alignment of NadA peptides evaluated by MATS. 12 peptide sequences evaluated by MATS were extracted from Neisseria PubMLST (NadA_peptide). Sequences are aligned against the peptide present in Bexsero® (8) and are grouped by NadA variant (NadA-1 variant – 1, 25, 100, 118, 137, 158; NadA-2/3 variant – 5, 6, 8, 142, 146).

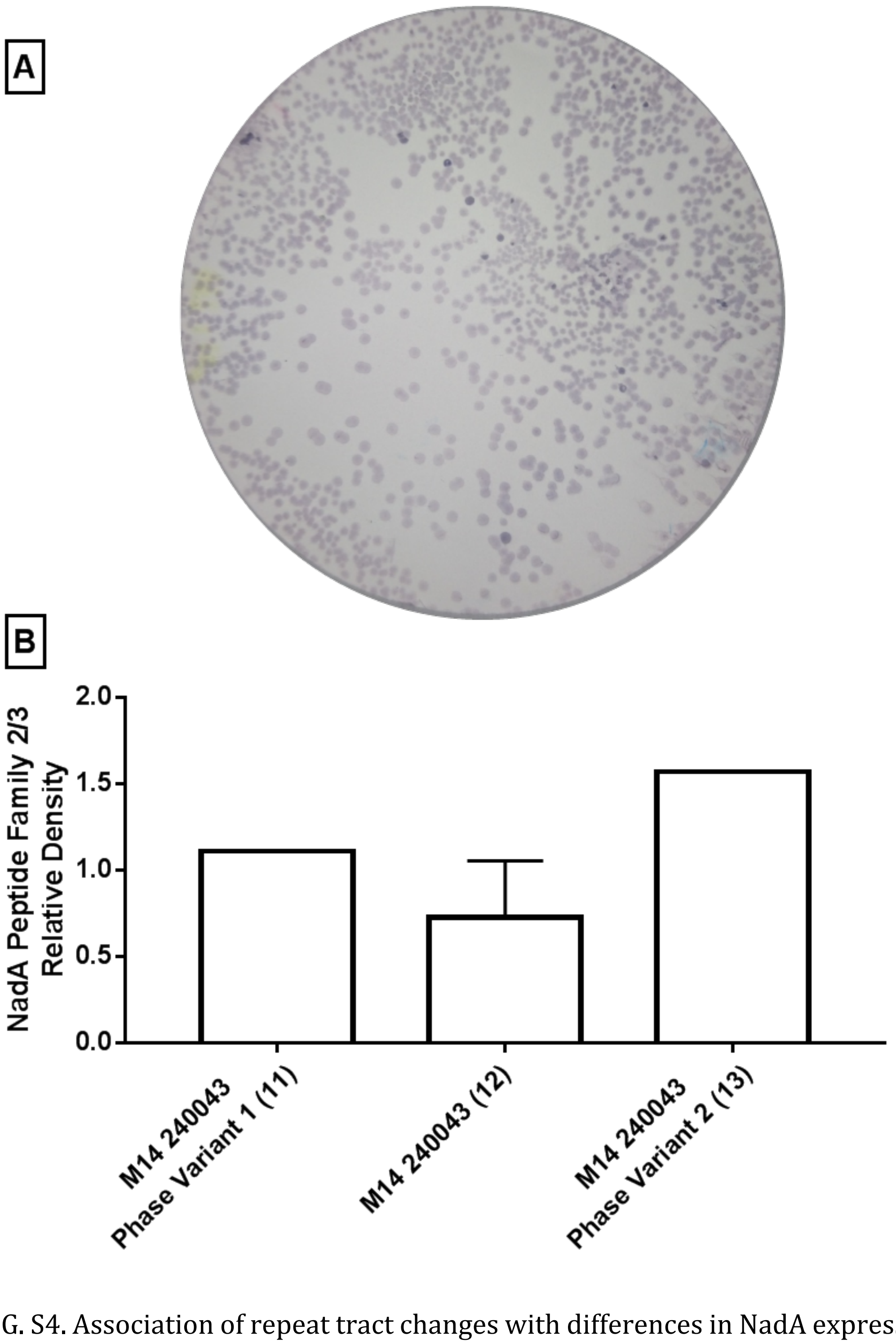

FIG. S4. Association of repeat tract changes with differences in NadA expression between isogenic colonies derived from the same isolate. Phase variants were isolated from colony immunoblots. Repeat numbers were confirmed by GeneScan analysis and sequencing. NadA expression was determined by Western blot and quantified by densitometry. NadA bands were normalised against Hsp60 bands and then relative density was assessed against M14 240602 (MenW). (A) Representative colony immunoblot of M14 240043. Darker staining represents phase variants suggesting a shift from low expression to high expression. (B) NadA expression in phase variants of M14 240043 with repeats ranging from 11-13. Numbers in brackets represent the 5'TAAA repeat number. M14 240043 (12) was for four replicates; M14 240043 (11) and M14 240043 (13) were one replicate.

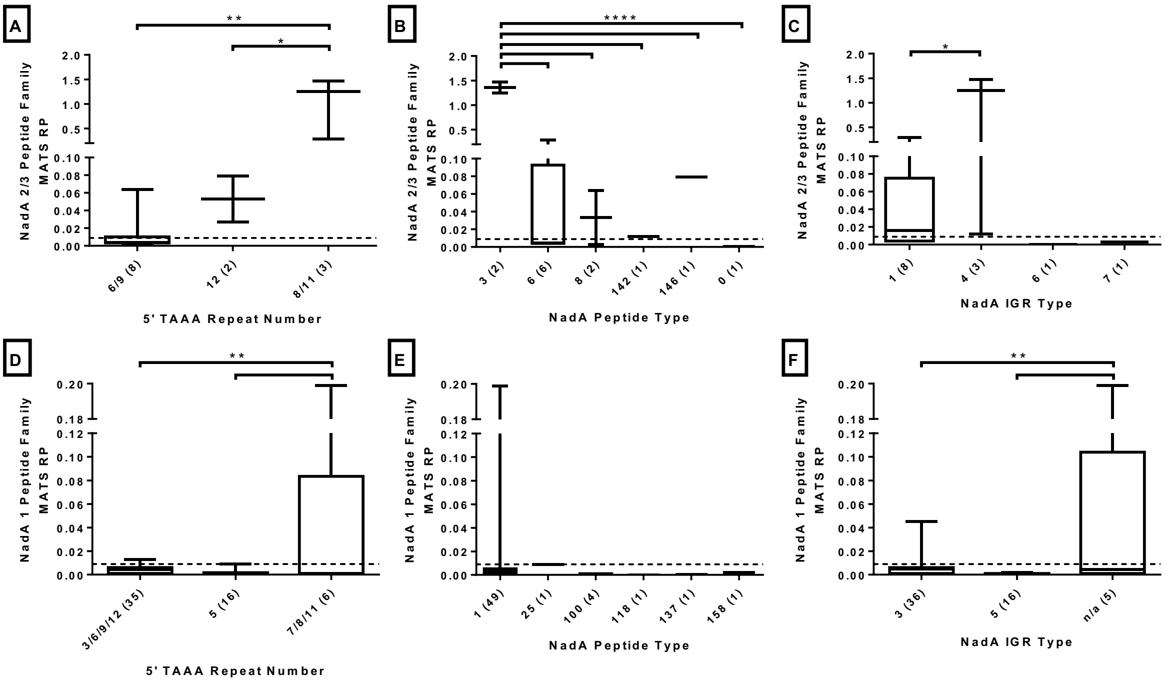

FIG. S5. Variant NadA-2/3 TAAA repeat number is predictive of high MATS RP scores. Subsets of MenB and MenW isolates expressing NadA 2/3 peptide family were analysed by the NadA MATS assay. Relative potency scores were compared against NadA repeat numbers, peptide and IGR types. Scores above or equal to the PBT of 0.009 were considered protective. Each symbol represents a different IGR type. Grey bars, above PBT; black bars, below PBT. Panel A,D, comparison of NadA expression groups based on repeat number against MATS RP scores. Panel B,E, comparison of NadA peptide type vs MATS RP scores, Panel C,F, comparison of NadA IGR type against MATS RP scores. Data was analysed by One Way ANOVA with Tukey's multiple comparison test; **, $p \leq 0.01$; *** $p \leq 0.001$.

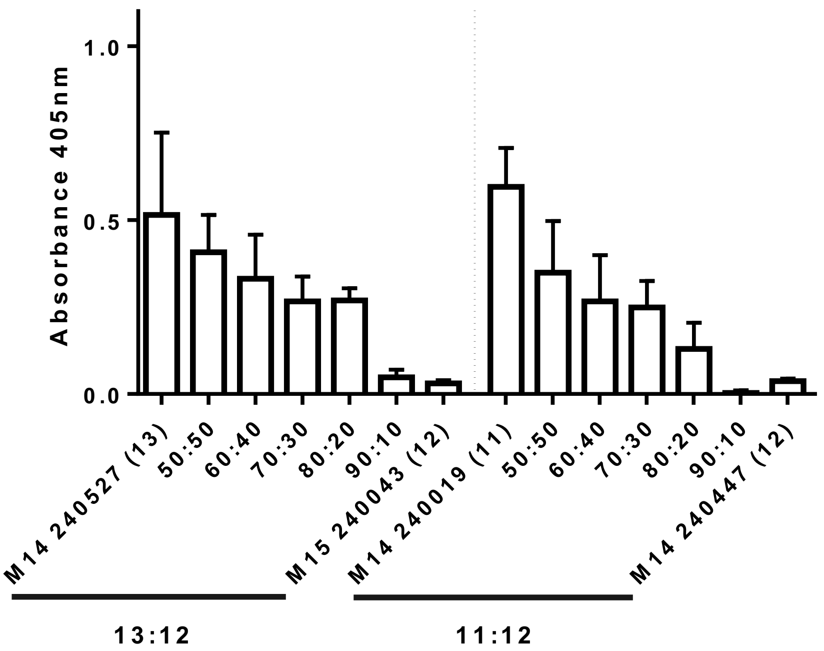

FIG. S6. Bias in NadA expression in heterogeneous mixes of meningococcal lysates observed by ELISA. NadA expression of various homogenous and heterogenous lysate mixes of MenW and MenB isolates expressing NadA-2/3 and NadA-1 peptide family were analysed by ELISA. Repeat numbers were confirmed by GeneScan analysis and sequencing. Several ratio mixes demonstrated the titrating effect of the presence of a high NadA expressing isolate. Numbers in brackets represent the 5'TAAA repeat number. Each bar represents three biological replicates. M14 240 527 and M15 240 043, both B:cc32:NadA peptide 1; M14 240 447 and M14 240 019, both W:cc11:NadA peptide 6.
