# Supplementary material for: Phase Variation of NadA in Invasive Neisseria meningitidis Isolates Impacts on Coverage Estimates for 4C-MenB, a MenB Vaccine
Source: J Clin Microbiol. 2018 Aug 27;56(9):e00204-18. doi: 10.1128/JCM.00204-18 (PMC6113495; doi:10.1128/JCM.00204-18)
Supplement: Supplemental file 2 [file zjm999096083s2.pdf]

TABLE S1. Comparison of NadA repeat numbers between genome sequence and GeneScan analysis data.

Isolate	Genogroup	IGR	Peptide	Genome Sequence	GeneScan Size	GeneScan Prediction
M14 240090	W	1	6	6	372.8	6
M14 240602	W	1	6	7	376	7
M12 240027	W	1	6	8	379.6	8
M11 240035	W	1	6	8	379.7	8
M12 240021	W	1	6	8	379.7	8
M14 240492	W	1	6	8	380.4	8
M11 240798	W	1	6	8	380.6	8
M12 240657	W	1	6	8	380.8	8
M11 240417	W	1	6	9	383.7	9
M14 240031	W	1	6	9	384	9
M12 240826	W	1	6	9	384.1	9
M13 240246	W	1	6	9	384.1	9
M13 240732	W	1	6	9	384.1	9
M11 240802	W	1	6	9	384.2	9
M11 240975	W	1	6	9	384.2	9
M12 240016	W	1	6	9	384.2	9
M12 240095	W	1	6	9	384.2	9
M12 240127	W	1	6	9	384.2	9
M12 240160	W	1	6	9	384.2	9
M12 240702	W	1	6	9	384.2	9
M12 240754	W	1	6	9	384.2	9
M11 240305	W	1	6	9	384.3	9
M12 240125	W	1	6	9	384.3	9
M12 240663	W	1	6	9	384.3	9
M14 240524	W	1	6	9	384.3	9
M14 240550	W	1	6	9	384.3	9
M14 240587	W	1	6	9	384.3	9
M11 240099	W	1	6	9	384.4	9
M12 240156	W	1	6	9	384.4	9

M12 240895	W	1	130	9	384.4	9
M14 240470	W	1	6	9	384.4	9
M11 240486	W	1	6	9	384.5	9
M12 240004	W	1	6	9	384.5	9
M12 240317[1]	W	1	6	12	387	10
M13 240028	W	1	6	10	388	10
M14 240548	W	1	6	10	388	10
M12 240640	W	1	6	10	388.2	10
M14 240258	W	1	6	10	388.2	10
M12 240133	W	1	6	10	388.4	10
M14 240555	W	1	6	11	391.8	11
M14 240019	W	1	6	11	391.9	11
M12 240067	W	1	6	11	392	11
M11 240067	W	1	6	12	395.6	12
M12 240337	W	1	6	12	395.6	12
M14 240257	W	1	6	12	395.6	12
M14 240351	W	1	6	12	395.6	12
M14 240500	W	1	6	12	395.6	12
M14 240581	W	1	6	12	395.6	12
M10 240514	W	1	6	12	395.7	12
M11 240427	W	1	6	12	395.7	12
M13 240283	W	1	6	12	395.7	12
M14 240283	W	1	6	12	395.7	12
M10 240817	W	1	6	12	395.8	12
M11 240168	W	1	6	12	395.8	12
M12 240144	W	1	6	12	395.8	12
M13 240600	W	1	6	12	395.8	12
M14 240142	W	1	6	13	395.8	12
M14 240304	W	1	6	12	395.8	12
M14 240447	W	1	6	12	395.8	12
M14 240573	W	1	6	12	395.8	12
M13 240446	W	1	6	12	395.9	12

M14 240495	W	1	6	12	395.9	12
M13 240015	W	1	6	12	396	12
M13 240436	W	1	6	12	396	12
M14 240043	W	1	6	12	396	12
M14 240468	W	1	6	12	396	12
M11 240057	W	1	6	12	396.1	12
M11 240953	W	1	6	12	396.1	12
M13 240681	W	1	6	13	399.5	13
M12 240240	W	1	6	13	399.7	13
M13 240705	W	1	6	17	402.9	14
M11 240403	W	1	6	14	403.2	14
M14 240178	W	1	6	14	403.6	14
M14 240517	W	1	6	15	407	15
M13 240531	W	1	6	15	407.2	15
M14 240448	W	1	6	15	407.2	15
M12 240324	W	1	6	15	407.4	15
M10 240821	W	1	6	15	407.5	15
M14 240389	W	1	6	15	407.5	15
M12 240774	W	1	6	15	407.6	15
M13 240025	W	1	6	15	407.6	15
M14 240204	W	1	6	16	411.2	16
M12 240898	W	1	6	17	411.5	16
M14 240180	W	1	6	16	411.5	16
M11 240726	W	1	6	17	411.7	16
M10 240671	W	1	6	17	411.8	16
M13 240269	W	1	6	17	411.8	16
M14 240624	W	1	6	17	415	17
M10 240701	B	2	0	7	382.9	9
M12 240277	B	3	1	5	368.2	5
M11 240016	B	3	100	6	372.2	6
M11 240175	B	3	1	6	372.2	6
M11 241019	B	3	1	6	372.2	6

M14 240383	B	3	1	6	372.4	6
M13 240017	B	3	1	9	383.9	9
M14 240480	B	3	1	12	395.7	12
M10 240684	B	4	3	9	383.7	9
M14 240014	B	5	1	5	368.3	5
M11 240723	B	8	21	8	379.6	8
M11 240002	B	8	21	9	383.8	9
M11 240109	B	8	21	12	395.7	12
M14 240282	W	17	6	15	407.4	15
M13 240672	W	n/d[2]	6	n/d	376.8	7
M13 240553	W	n/d	6	n/d	376.9	7
M14 240532	W	n/d	6	n/d	379.6	8
M14 240562	W	n/d	6	n/d	380.8	8
M14 240126	W	n/d	6	11	392.2	11
M14 240026	W	n/d	6	12	395.7	12
M14 240478	W	n/d	6	n/d	395.7	12
M14 240556	W	n/d	6	12	395.8	12
M13 240545	W	n/d	6	n/d	395.9	12
M14 240082	W	n/d	6	13	399.9	13
M14 240072	W	n/d	6	15	407.3	15
M14 240054	W	n/d	6	n/d	411.1	16
M13 240491	W	n/d	6	n/d	411.3	16
M14 240496	W	n/d	6	n/d	411.8	16
M14 240511	W	n/d	6	n/d	411.8	16
M14 240485	W	n/d	6	n/d	411.9	16
M14 240052	W	n/d	6	19	427.1	20

[1]Greyed out sections signify a mismatch between whole genome sequencing data and GeneScan fragment analysis. [2]n/d no data, this signifies an isolate where the *nadA* IGR or the 5’TAAA repeat length could not be determined from the whole genome sequence data.

TABLE S2. Genomic information for NadA peptide and IGR sequences for isolates tested by ELISA and Western blot analyses.

Isolate	Genogroup	Clonal Complex	NadA Peptide Family	NadR Peptide	NadA Peptide	IGR	Repeat Tract
M14 240090	W	cc11	2/3	1	6	1	6
M10 240650	B	n/d[1]	2/3	1	8	1	6
M14 240602	W	cc11	2/3	1	6	1	7
M14 240492	W	cc11	2/3	1	6	1	8
M11 240798	W	cc11	2/3	1	6	1	8
M14 240470	W	cc11	2/3	1	6	1	9
M14 240031	W	cc11	2/3	1	6	1	9
M10 240684	B	cc11	2/3	1	3	4	9
M12 240754	W	cc11	2/3	1	6	1	9
M15 240003	B	cc269	2/3	1	8	7	9
M07 241012	B	cc11	2/3	n/d[2]	142	4	9
M14 240548	W	cc11	2/3	1	6	1	10
M14 240019	W	cc11	2/3	1	6	1	11
M08 240227	B	cc11	2/3	1	3	4	11
M14 240043	W	cc11	2/3	1	6	1	12

M14 240447	W	cc11	2/3	1	6	1	12
M15 240281	B	n/d	2/3	1	146	1	12
M14 240082	W	cc11	2/3	1	6	n/d[3]	13
M14 240282	W	cc11	2/3	1	6	17	15
M14 240204	W	cc11	2/3	1	6	1	16
M14 240485	W	cc11	2/3	1	6	n/d	17
M14 240624	W	cc11	2/3	1	6	1	17
M14 240052	W	cc11	2/3	1	6	n/d	20
M12 240277	B	cc32	1	2	1	3	5
M14 240014	B	cc32	1	2	1	5	5
M14 240528	B	cc32	1	2	1	5	5
M11 240016	B	cc32	1	4	100	3	6
M11 240175	B	cc32	1	2	1	3	6
M11 241019	B	cc32	1	1	1	3	6
M14 240383	B	cc32	1	2	1	3	6
M14 240560	B	n/d	1	2	1	n/d	6
M14 240576	B	cc32	1	2	1	3	6
M14 240467	B	cc32	1	2	1	3	8

M14 240620	B	cc32	1	13	100	n/d	8
M13 240017	B	cc32	1	2	1	3	9
M07 240868	B	cc32	1	n/d	1	3	9
M14 240480	B	cc32	1	2	1	3	12
M15 240001	B	cc32	1	2	1	3	12
M15 240043	B	cc32	1	2	1	n/d	12
M15 240527	B	cc32	1	2	1	n/d	13
M11 240723	B	n/d	4/5	1	21	8	8
M11 240002	B	n/d	4/5	1	21	8	9
M11 240109	B	cc32	4/5	4	21	8	12
M15 240004	B	cc1157	2/3	1	0	6	6
M10 240701	B	cc213	4/5	1	0	2	7
R001:*ΔnadA*	W	cc11	2/3	1	Δ	1	12

Grey signifies those isolates tested with the MATS assay. [1] "n/d" signifies that no clonal complex was provided within the MRF-MGL. [2] "n/d" signifies the NadR peptide could not be determined from whole genome sequencing. [3] "n/d" signifies the NadA IGR could not be determined from whole genome sequencing data.

TABLE S3. Comparison of MATS values and repeat number for NadA

Isolate	Geno-group	NadA Variant	Clonal Complex	NadA Peptide	NadR Peptide [1]	IGR	Repeat Tract	Predicted Expression – Repeat Tract	RP values
M10 240650	B	2/3	n/d[3]	8	1	1	6	Low	0.0640[2]
M15 240004	B	2/3	cc1157	0	1	6	6	Zero	0.0002
M15 240003	B	2/3	cc269	8	1	7	9	Low	0.0030
M07 241012	B	2/3	cc11	142	n/d[4]	4	9	Low	0.0120
M07 241076	B	2/3	cc11	3	n/d	4	11	High	1.4740
M08 240227	B	2/3	cc11	3	1	4	11	High	1.2480
M15 240281	B	2/3	n/d	146	1	1	12	Low	0.0790
M11 240798	W	2/3	cc11	6	1	1	8	High	0.2900
M11 240417	W	2/3	cc11	6	1	1	9	Low	0.0040
M11 240802	W	2/3	cc11	6	1	1	9	Low	0.0050
M12 240016	W	2/3	cc11	6	1	1	9	Low	0.0040
M12 240754	W	2/3	cc11	6	1	1	9	Low	0.0040
M11 240427	W	2/3	cc11	6	1	1	12	Low	0.0270
M16 240221	B	1	cc32	1	2	3	3	Low	0.0050
M14 240513	B	1	cc32	100	2	3	5	Low	0.0002
M14 240528	B	1	cc32	1	2	5	5	Low	0.0020

M15 240020	B	1	n/d	1	1	n/d[5]	5	Low	0.0010
M15 240065	B	1	cc32	100	4	3	5	Low	0.0006
M07 240732	B	1	cc32	1	n/d	5	5	Low	0.0009
M07 240927	B	1	cc32	1	n/d	5	5	Low	0.0000
M07 241099	B	1	cc32	1	n/d	5	5	Low	0.0010
M07 241136	B	1	cc32	1	n/d	5	5	Low	0.0020
M08 240022	B	1	cc32	25	n/d	3	5	Low	0.0090
M08 240112	B	1	cc32	1	n/d	5	5	Low	0.0009
M08 240157	B	1	cc32	1	n/d	5	5	Low	0.0020
M08 240247	B	1	n/d	1	n/d	5	5	Low	0.0000
M08 240297	B	1	cc32	1	n/d	5	5	Low	0.0010
M08 240464	B	1	cc32	1	n/d	5	5	Low	0.0000
M08 240495	B	1	cc32	1	n/d	5	5	Low	0.0000
M08 240503	B	1	cc32	1	n/d	5	5	Low	0.0000
M14 240062	B	1	cc32	137	1	3	6	Low	0.0002
M14 240383	B	1	cc32	1	2	3	6	Low	0.0070
M14 240560	B	1	n/d	1	2	n/d	6	Low	0.0090
M14 240576	B	1	cc32	1	2	3	6	Low	0.0050

M14 240591	B	1	cc32	158	1	3	6	Low	0.0020
M14 240598	B	1	cc32	1	2	3	6	Low	0.0060
M14 240623	B	1	cc32	118	1	3	6	Low	0.0001
M15 240069	B	1	cc32	100	n/d	3	6	Low	0.0010
M15 240113	B	1	cc32	1	2	3	6	Low	0.0055
M15 240142	B	1	cc32	1	2	3	6	Low	0.0045
M15 240279	B	1	cc32	1	2	3	6	Low	0.0060
M15 240304	B	1	n/d	1	2	3	6	Low	0.0070
M15 240425	B	1	cc32	1	2	3	6	Low	0.0050
M07 240673	B	1	cc32	1	n/d	3	6	Low	0.0040
M07 241039	B	1	cc32	1	n/d	3	6	Low	0.0030
M07 241065	B	1	cc32	1	n/d	5	6	Low	0.0000
M07 241114	B	1	cc32	1	n/d	3	6	Low	0.0050
M08 240081	B	1	cc32	1	n/d	3	6	Low	0.0040
M08 240133	B	1	cc32	1	n/d	3	6	Low	0.0000
M08 240177	B	1	cc32	1	n/d	3	6	Low	0.0030
M08 240202	B	1	cc32	1	n/d	3	6	Low	0.0060
M08 240404	B	1	cc32	1	n/d	3	6	Low	0.0050

M08 240494	B	1	cc32	1	n/d	3	6	Low	0.0040
M08 240525	B	1	cc32	1	n/d	3	6	Low	0.0050
M15 240574	B	1	n/d	1	2	3	6	Low	0.0050
M08 240034	B	1	cc32	1	n/d	5	7	Low	0.0000
M08 240225	B	1	cc32	1	n/d	5	7	Low	0.0000
M11 240406	B	1	cc32	1	2	5	7	Low	0.0009
M14 240467	B	1	cc32	1	2	3	8	High	0.0450
M14 240620	B	1	cc32	100	13	n/d	8	High	0.0010
M07 240868	B	1	cc32	1	n/d	3	9	Low	0.0000
M08 240063	B	1	cc32	1	n/d	3	9	Low	0.0010
M08 240151	B	1	cc32	1	n/d	3	9	Low	0.0000
M08 240153	B	1	cc32	1	n/d	3	9	Low	0.0010
M08 240375	B	1	cc32	1	n/d	3	9	Low	0.0000
M12 240803	B	1	cc32	1	2	3	12	Low	0.0100
M14 240480	B	1	cc32	1	2	3	12	Low	0.0110
M15 240001	B	1	cc32	1	2	3	12	Low	0.0130
M15 240043	B	1	cc32	1	2	n/d	12	Low	0.0045
M14 240527	B	1	cc32	1	2	n/d	13	High	0.199

M14 240372	B	4/5	cc213	0[6]	2	2	12	Zero	0.0001
M15 240067	B	4/5	cc213	0	2	2	12	Zero	0.0002
M15 240038	B	4/5	cc213	0	2	2	14	Zero	0.0000

[1]NadR peptide derived from *nadR* alleles extracted from the MRF-MGL database. [2]Greyed out boxes signify an RP greater than or equal to the PBT (0.009). [3] "n/d" signifies that no clonal complex was provided within the MRF-MGL. [4]"n/d" signifies when the NadR peptide could not be determined from whole genome sequencing data. [5]"n/d" signifies when the NadA IGR could not be determined from whole genome sequence data. [6]All NadA-4/5 isolates tested are frame-shifted due to an intragenic polyC tract (M14 240372, 10; M15 240067, 10; M15 240038, 10).
